# Do adolescents understand the items of the European Health Literacy Survey Questionnaire (HLS-EU-Q47) – German version? Findings from cognitive interviews of the project “Measurement of Health Literacy Among Adolescents” (MOHLAA) in Germany

**DOI:** 10.1186/s13690-018-0276-2

**Published:** 2018-07-10

**Authors:** Olga Maria Domanska, Christiane Firnges, Torsten Michael Bollweg, Kristine Sørensen, Christine Holmberg, Susanne Jordan

**Affiliations:** 1 Department of Epidemiology and Health Monitoring, Robert Koch Institute, Berlin, Germany; 2 Centre for Prevention and Intervention in Childhood and Adolescence (CPI), Bielefeld University, Bielefeld, Germany; 3 Global Health Literacy Academy, Risskov, Denmark; 4 Institute of Public Health, Charité – Universitätsmedizin Berlin, corporate member of Freie Universität Berlin, Humboldt - Universität zu Berlin and Berlin Institute of Health, Berlin, Germany; 5 Institute of Social Medicine and Epidemiology, Medical School Brandenburg Theodor Fontane, Brandenburg/Havel, Germany

**Keywords:** Health literacy, Measurement, HLS-EU-Q, Qualitative, Cognitive interviewing, Adolescents, Germany

## Abstract

**Background:**

In Germany, there are no measurement tools to assess the general health literacy of adolescents. The aim of the study “Measurement of Health Literacy Among Adolescents” (MOHLAA) is to develop such a tool for use among adolescents aged 14–17. The German version of the European Health Literacy Survey Questionnaire (HLS-EU-Q47-GER) served as a blueprint for the development of the tool. The present study examined the extent to which the HLS-EU-Q47-GER can be applied to the measurement of general health literacy in adolescents.

**Methods:**

The applicability of the HLS-EU-Q47-GER for adolescents was tested qualitatively using cognitive interviewing (CI). Purposive sampling was used to achieve an equal distribution of participants regarding age groups, educational backgrounds and gender. CI was standardized on the basis of an interview guide. Verbal probing and the retrospective think-aloud technique were applied. The interviews were audio-recorded, transcribed and analyzed using the criteria of theory-based analysis, which were derived from the model of cognitive processes. The analysis focused on identifying terms and questions that were difficult to understand and on scrutinizing the extent to which the content of the items is appropriate for assessing adolescents’ health literacy.

**Results:**

Adolescent respondents were unfamiliar with some terms of the HLS-EU-Q47-GER or provided heterogeneous interpretations of the terms. They had limited or no experience regarding some health-related tasks in health care and disease prevention that are addressed by HLS-EU-Q-items. A few items seemed to be too “difficult” to answer due to a high abstraction level or because they lacked any reference to the everyday lives of youth. Despite comprehension problems with some of the HLS-EU items, the respondents assessed the covered health-related tasks as “very easy” or “fairly easy”. CI stressed the importance of interpersonal agents, especially parents, in helping adolescents understand and judge the reliability of health information.

**Conclusions:**

The results of CI indicated that the applicability of the HLS-EU-Q47-GER to the measurement of general health literacy among adolescents aged 14–17 is limited. In order to prevent biased data, some items of the questionnaire should be adjusted to adolescents’ state of development and experiences with health care and disease prevention.

**Electronic supplementary material:**

The online version of this article (10.1186/s13690-018-0276-2) contains supplementary material, which is available to authorized users.

## Background

The health literacy of children and adolescents is receiving increasing attention from scholars and practitioners. This growing interest is based on the assumption that better health literacy skills at a young age will improve health outcomes in adult life [1, 2]. The development of such skills among children and adolescents is even regarded as an opportunity to empower this vulnerable group to be more “engaged” and “productive” citizens [3]. Health literacy is commonly described as the set of health-related knowledge and abilities that enable a person to use resources and to make healthy choices in everyday life [4–6]. In this broad and comprehensive definition of health literacy, there is no restriction to specific aspects of health literacy, such as the use of health information with respect to specific health-related topics (e.g., diabetes literacy, mental health literacy) or contexts or domains (e.g., health literacy in healthcare, functional health literacy) [7, 8]. Health literacy is depicted as a skill that changes and develops through the life course [5]. Hence, acquiring health literacy at a young age may be a base for health-related quality of life as well as a promising approach to disease prevention and health promotion [1, 9].

Adolescence is regarded as a “transitional” stage that involves physical, emotional, and cognitive changes [10, 11]. Young people have to manage different developmental tasks, such as developing their own values and norms or establishing their gender identity [12]. At the same time, this period of life is characterized by an increase in risky behavior (e.g., unprotected sexual activity, dangerous driving, illegal substance use) and perception of vulnerability [13, 14]. In this regard, many studies show that adolescents’ health behaviors are linked to health behaviors and health outcomes in later life [15–18]. In addition, in the age of digital technology and easy access to information, young people are constantly using online services, social media, and mobile applications, and they increasingly turn to digital media to answer their health-related questions [19, 20].

The results of the review by Fleary et al. regarding adolescent health literacy and health behaviors suggest that there is a meaningful relationship between health literacy and adolescents’ health behaviors. However, this relationship was found in cross-sectional studies in which specific domains of health literacy such as functional and media health literacy were assessed [21]. Little is known about the distribution of general health literacy in adolescents and whether there is a link to their health behaviors. Thus far, no longitudinal studies have examined the predictive value of certain levels of health literacy in childhood and/or adolescence for health outcomes in later life. Accordingly, most statements about the relevance of the early promotion of health literacy are either theoretical or draw on evidence from related fields of research. Reviews of health literacy concepts and measurements in children and adolescents have noted that even though research in this field is increasing, clear definitions and measures – including comprehensive measurement tools – are still in development [9, 22, 23]. Furthermore, Okan et al. specify a need for health literacy measurements that are better suited to the specific health literacy needs of children and adolescents [22].

In Germany, we found only a few studies on adolescents’ health literacy; these focused on the measurement of specific health literacy domains, such as health-related knowledge [24] or critical health literacy [25, 26], or they targeted certain age groups of adolescents: 9–13 year-olds [27], 15–29 year-olds [28] or a specific subgroup such as educationally alienated young people [29]. In the project “Measurement of Health Literacy Among Adolescents (MOHLAA),” we aim to develop a self-administered tool to measure the comprehensive concept of heath literacy (general health literacy) among adolescents aged 14 to 17. Because developmental stages differ throughout adolescence from 12 to 19 years of age, the development of a single instrument adequate for all age groups might not be feasible [30]. Hence, we focused here on young people in mid-adolescence, as they become more independent in making their own decisions and their risky behavior patterns increase [31, 32].

We chose the German version of the European Health Literacy Survey Questionnaire (HLS-EU-Q47-GER) as a blueprint for our tool. The HLS-EU-Q47 was developed and validated by the HLS-EU consortium for the comparative assessment of heath literacy among eight European countries [33]. More details about the conceptual model of the HLS-EU-Q47, the development of the questionnaire, its translation, and its validation are provided by Sørensen et al. [5, 34, 35] and the European Health Literacy Project Consortium [36]. We took the HLS-EU-Q47-GER as a starting point for two reasons. Firstly, this questionnaire is based on a comprehensive conceptual model of health literacy. It focuses not only on health-related tasks in the health care setting but also on such tasks as they relate to health promotion and disease prevention, which are essential target domains for interventions in this age group. Secondly, the HLS-EU-Q47 was already applied in a large international sample – the European Health Literacy Survey, which had 8000 participants, including 1000 from the federal state of North Rhine-Westphalia, Germany. By the time the MOHLAA project started in 2015, the HLS-EU-Q47-GER and its short form (HLS-EU-Q16) had been applied in other studies in Germany [28, 37–39].

An in-depth examination of the applicability of the HLS-EU-Q47-GER among adolescents was deemed necessary for several reasons. The HLS-EU questionnaire was designed according to the Eurobarometer standards for surveys (face-to-face) in adult populations, defined as aged 15 years and above. Although some adolescents (aged 15+) participated in the field pretesting of the HLS-EU-Q47 in Ireland and the Netherlands, no published information is available on the acceptance and applicability of the questionnaire in this age group [35]. We wanted to examine whether the instrument could be applied as a self-administered paper-and-pencil questionnaire if respondents do not have a chance to ask an interviewer for help or explanations. Findings from the Heath Literacy Youth-Study in Austria (15 year-olds) [40] and from a pretest of the “German Health Interview and Examination Survey for Children and Adolescents” (14–17 year-olds) [41] indicate, based on the analysis of missing values and the distribution of responses, that some items of the HLS-EU-Questionnaire may be too difficult to understand for adolescents. The researcher of the Austrian Youth-Study suspected that for adolescents, the difficulty of some items may be due to unknown and incomprehensible terms, or to insufficient experience and knowledge with regard to health care [40]. However, the authors do not report in detail the specific terms that people in this age group find difficult to understand, which may be a reason for the observed numbers of missing responses. Accordingly, no in-depth information is currently available on the specific problems that can arise when using the HLS-EU questionnaire among adolescents. Thus, there is a lack of evidence with regard to whether and how the questionnaire can be revised so that it is appropriate for younger participants. Finally, a measurement tool applicable to adolescents should respond to their unique health-related needs and characteristics by distinguishing children and adolescents from the general adult population (cf. Rothmann et al. [42]) and by taking into account adolescents’ conceptualization of health and their health-related knowledge, as explored in other studies of youth [43, 44].

The present study aims to investigate the applicability of the HLS-EU-Q47-GER for measuring the general health literacy of adolescents aged 14–17. The following research questions were examined: (1) To what extent do adolescents comprehend the items of the HLS-EU-Q47-GER? (2) Does the questionnaire consider the specific characteristics of adolescents, e.g., their cognitive abilities and their experience with health-related information?

## Methods

### Sampling and study population

We recruited respondents using a snowball approach according to a purposive sampling strategy [45]. Sampling criteria included age (14–17-year-olds in 4 age groups), educational background (in high school vs. not in high school), and gender (girls and boys). The category “not in high school” included all types of lower- and middle-secondary level schools existing in Berlin’s education system at the time.

The sample size (*n* = 20) was chosen based on recommendations for cognitive interviews [46–48]. To recruit the purposive sample, we approached a variety of different venues to reach respondents with a variety of schooling backgrounds. For example, we sought adolescents from youth clubs, girls’ clubs and sports clubs in socially disadvantaged city districts and from our research institute’s employees.

### Cognitive interviewing

Cognitive interviewing (CI) is an approach that is used to evaluate sources of response error in survey questionnaires [49]. This approach is recommended for use in the early phase of questionnaire development in order to gain insights into the cognitive processes of responding [46, 48–51]. The aims of our CI were (1) to assess the comprehensibility of questions and terms; (2) to explore which personal experiences the given answers were based upon and what type of knowledge adolescents applied while answering the HLS-EU-Q47-GER items.

Mostly, verbal probes (specific and general probes) and retrospective think-aloud techniques [51, 52] were used. General probes after each of the three HLS-EU-Q47-GER subscales asked for non-specific information, e.g., “*How easy to answer were the questions in this section for you*?” or “*Which of the questions was the most difficult?”.* The general probe “*Could you answer the questions based on your experiences*?” aimed to examine the extent to which adolescents answered hypothetically. In turn, specific probes aimed to investigate adolescents’ understanding of certain terms, the content of items, or whether adolescents’ specific experiences were applicable, e.g.: “*What does the term ‘health risks’ mean to you?”, or “What health risks come to your mind when you think about your friends?”*. When it became apparent during the interview that there was a problem with the questionnaire, e.g., when adolescents spontaneously asked for clarification or they took noticeably longer to give an answer, the interviewers applied ad hoc probes, e.g., “*Is there something that you didn’t understand in this question?”*

### Data collection and procedure

The 20 interviews were carried out from December 2015 to March 2016 at the Robert Koch Institute, Berlin, Germany. The interviews lasted between 55 and 110 min. (70 min. on average). Cognitive interviews were conducted in a standardized manner by two interviewers (one 24-year-old female and one 26-year-old male interviewer), who used an interview guide developed by the research team. The interview guide included the detailed interview procedure and the items with their corresponding probes. Sixteen items were tested closely; these items are shown in bold in Table 1, which contains the original HLS-EU-Q items in English. The HLS-EU-Q47-GER (Additional file 1: Table S1) is a translation of the English version that has been verified within the scope of the European Health Literacy Survey [35]. Both versions of the HLS-EU-Q47 were provided by the developers of the instrument. We selected the items based on the findings of the Austrian Youth Study [27] and on theoretical assumptions about the cognitive abilities of adolescents in relation to the item difficulty and complexity [11]. Accordingly, we tested items with a proportion of missing values higher than the 5% observed in the Austrian study (e.g., items 1, 11, 18) and items that included sophisticated, abstract terms as “health risks”, “political changes”, “effort to promote your health”, etc., or inquired about complex, cognitively demanding issues (e.g., item 42 “…judge how housing conditions help you to stay healthy”). Due to time constraints for cognitive interviews, we tested a sample of items that refer to specific domains of the HLS-EU questionnaire; therefore, we report the findings referring only to those domains.

**Table 1:** The English version of European Health Literacy Survey Questionnaire (HLS-EU-Q47)

Introduction text		On a scale from very easy to very difficult, how easy would you say it is to:
Response categories		1 Very difficult; 2 Fairly Difficult; 3 Fairly Easy; 4 Very Easy
No.	HLS-EU-Q dimension	**Subscale: HEALTH CARE**
**1** ^a^	Access	**…find information about symptoms of illnesses that concern you?**
2		…find information on treatments of illnesses that concern you?
**3** ^a^		**…find out what to do in case of a medical emergency?**
4		…find out where to get professional help when you are ill? *(Instructions: such as doctor, pharmacist, psychologist)*
5	Understand	…understand what your doctor says to you?
**6** ^a^		**…understand the leaflets that come with your medicine?**
**7** ^a^		**…understand what to do in a medical emergency?**
8		…understand your doctor’s or pharmacist’s instructions on how to take a prescribed medicine?
9	Appraise	…judge how information from your doctor applies to you?
10		…judge the advantages and disadvantages of different treatment options?
**11** ^a^		**…judge when you may need to get a second opinion from another doctor?**
**12** ^a^		**…judge if the information about illness in the media is reliable?** *(Instructions: TV, Internet or other media)*
13	Apply	…use information the doctor gives you to make decisions about your illness?
14		…follow the instructions on medication?
15		…call an ambulance in an emergency?
16		…follow instructions from your doctor or pharmacist?
		**Subscale: DISEASE PREVENTION**
17	Access	…find information about how to manage unhealthy behaviour such as smoking, low physical activity and drinking too much?
**18** ^a^		**…find information on how to manage mental health problems like stress or depression?**
19		…find information about vaccinations and health screenings that you should have? *(Instructions: breast exam, blood sugar test, blood pressure)*
20		…find information on how to prevent or manage conditions like being overweight, high blood pressure or high cholesterol?
21	Understand	…understand health warnings about behaviour such as smoking, low physical activity and drinking too much?
22		…understand why you need vaccinations?
**23** ^a^		**…understand why you need health screenings?** *(Instructions: breast exam, blood sugar test, blood pressure)*
24	Appraise	…judge how reliable health warnings are, such as smoking, low physical activity and drinking too much?
25		…judge when you need to go to a doctor for a check-up?
26		…judge which vaccinations you may need?
27		…judge which health screenings you should have? *(Instructions: breast exam, blood sugar test, blood pressure)*
**28** ^a^		**…judge if the information on health risks in the media is reliable?** *(Instructions: TV, Internet or other media)*
29	Apply	…decide if you should have a flu vaccination?
30		…decide how you can protect yourself from illness based on advice from family and friends?
31		…decide how you can protect yourself from illness based on information in the media? *(Instructions: Newspapers, leaflets, Internet or other media?)*
		**Subscale: HEALTH PROMOTION**
32	Access	…find information on healthy activities such as exercise, healthy food and nutrition?
**33** ^a^		**…find out about activities that are good for your mental well-being?** *(Instructions: meditation, exercise, walking, pilates* etc.*)*
**34** ^a^		**…find information on how your neighborhood could be more health-friendly?** *(Instructions: Reducing noise and pollution, creating green spaces, leisure facilities)*
**35** ^a^		**…find out about political changes that may affect health?** *(Instructions: legislation, new health screening programmes, changing of government, restructuring of health services* etc.*)*
**36** ^a^		**…find out about efforts to promote your health at work?**
37	Understand	…understand advice on health from family members or friends?
38		…understand information on food packaging?
39		…understand information in the media on how to get healthier? *(Instructions: Internet, newspapers, magazines)*
40		…understand information on how to keep your mind healthy
41	Appraise	…judge where your life affects your health and well-being? *(Instructions: Your community, your neighbourhood)*
**42** ^a^		**…judge how your housing conditions help you to stay healthy?**
43		…judge which everyday behavior is related to your health? *(Instructions: Drinking and eating habits, exercise* etc.*)*
44	Apply	…make decisions to improve your health?
45		…join a sports club or exercise class if you want to?
**46** ^a^		**…influence your living conditions that affect your health and wellbeing?** *(Instructions: Drinking and eating habits, exercise* etc.*)*
**47** ^a^		**…take part in activities that improve health and well-being in your community?**

^a^Items tested with specific probes are shown in bold. HLS-EU-Q dimensions are not shown to the respondents

 First, the respondents filled out one subscale of the HLS-EU-Q47-GER. Then, they were queried on the whole subscale with general probes, followed by specific probes referring to selected items. This procedure was performed three times, as the HLS-EU-Q47-GER consists of three subscales (Additional file 1: Table S1).

### Data analysis

The interviews were audio-recorded, transcribed and entered into a standardized data analysis sheet by the interviewers. Interviewers’ notes were also examined in order to add details to the transcripts. The data were structured according to the recommendation of Prüfer and Rexroth [48]: for each HLS-EU item, a case-specific list of all respondents’ statements with the probes was compiled. Then, the data were analyzed by one coder (OD) based on seven analysis criteria. These criteria, which are presented in Table 2, were derived from the general cognitive model of response processes formulated by Tourangeau [53].

**Table 2:** Standardized analysis criteria applied to the cognitive interviews of the MOHLAA study in Germany (12/2015–03/2016)

Cognitive processes by Tourangeau	Criterion	Corresponding research questions
Comprehension of the item wording	**C1** Sentence structure/grammar	Is the sentence syntax of the item clear? Is the wording of the item immediately/easily understood?
Comprehension of the intention of the question	**C2** Comprehensibility of item content	Is the item understood as intended? Was the item interpreted similarly by different respondents?
Comprehension of the meaning of the terms	**C3** Understanding of terms/hints	Could a proper definition of the term be given? Was a given definition unambiguous? Were hints in the item comprehensible and familiar?
Retrieval of relevant information from memory	**C4** Difficulty	Was the item assessed as “easy” or as “difficult” to answer? Why was it assessed as “difficult”?
	**C5** Experience/knowledge	What type of knowledge and experience were recalled? Is the given answer based on any experience or related to an abstract idea/concept?
Decision process, Motivation, sensitivity, social desirability	**C6** Reliability of the response	Does the reported justification of the given response suggest that the item evokes a tendency of social desirability? Which motivation might underlie the given answer?
Response process	**C7** Accordance of the formal response category with an internal ascertained response category.	Can respondent find his/her answer option on the response category scale?

Depending on the occurrence of comprehension problems and on the applied probes, the extent to which these criteria were satisfied for each HLS-EU item was examined. The number of unanswered items (missing values) with reported justifications by respondents was considered an additional possible indication of comprehension problems or lack of relevance of the item. Based on the Framework Method described by Collins [54] and Gale et al. [55], we categorized the results of single-item analysis into key themes illustrating the main limitations of the HLS-EU-Q47-GER when used among adolescent respondents.

In order to ensure the “objectivity” [56] of the CI (with respect to application, analysis, and interpretation) different procedures were implemented. The interviewers attended a 2-day training and received feedback on their performance during the training and the field phase from two project researchers (OD/CF). The completeness and accuracy of the transcripts were verified by one member of the research team (CF), who independently coded parts of the transcripts. The entire research team (CF/SJ/OD) discussed the results of the analysis and the implications for the HLS-EU-Q47-GER.

## Results

The sample characteristics are presented in Additional file 2: Table S2. Participants from different age groups and genders were represented. We interviewed a total of *n* = 20 adolescents attending various school types. School types were categorized as (1) upper secondary education level (high school; *n* = 6) and (2) lower and middle secondary education levels (*n* = 14).

### Single-item findings

Detailed results for each item are summarized in Additional file 3: Table S3. 19 HLS-EU-Q47-GER items seemed to work as intended, and participants did not make any relevant remarks. However, due to the time constraints and the research questions, only two of those items were tested in-depth with specific probes. Problems related to **comprehensibility** were identified for 21 items. More specifically, we found items that included an unknown/ambiguous term (items 1, 11, 17, 18, 19, 20, 23, 33–36, and 47), had unclear wording or meaning (items 7, 9, 10, 13 and 46), or had a challenging level of abstraction (items 35, 41–43). Moreover, problems were identified concerning the **relevance of items** for the age group: respondents either lacked the required experience to give a reasoned answer (items 6, 10–13, 18 and 40), or items were not applicable to the age group (items 22, 23, 26, 27 and 29). Items with the largest proportion of missing values (3–4 of 20), potentially indicating both of these problems, were identified in the health promotion subscale (items 34, 41, 43 and 47). Issues relating to the comprehensibility and relevance of items were also observed in combination. For example, participants did not understand the term “*Unterstützungsmöglichkeiten”* (possibilities for support) in the context of mental health, and they could not relate to the item, as the majority of respondents reported that they had never had to address mental health problems.

Additional file 3: Table S3 includes conclusions we derived from the results that may be useful for researchers interested in the adaption of the HLS-EU-Q for adolescents. These conclusions go beyond of the scope of the primary research question and are therefore not discussed in detail.

### General findings concerning comprehensibility and relevance for adolescents

Data analysis revealed five dominant themes: (1) Unfamiliar terms or ambiguous interpretations of terms (comprehensibility); (2) Challenging level of abstraction (comprehensibility); (3) Lack of experience and knowledge (relevance); (4) Mapping the response options, and (5) Importance of parents (relevance). These findings are presented in detail in the following.

### Unfamiliar terms or ambiguous interpretations of terms (comprehensibility)

Adolescent respondents were unfamiliar with certain terms in the HLS-EU-Q47-GER. This became evident when participants were asked to explain, in their own words, the meaning of a term or to provide an example for this term. For instance, four respondents reported not knowing the term “*Krankheitssymptome”* (symptoms of illnesses; item 1), although most respondents (15/20) were able to define it and give appropriate examples. One person was not able to describe the term correctly. However, only two of the four respondents who did not know the term left the question unanswered. A further example for a difficult term was “*Gesundheitsrisiken”* (health risks; items 20 and 28). One respondent interpreted the term incorrectly as the risk of having any symptoms or disorders whilst being ill (ID_11)^1^: “risk of losing blood because of having a certain disease”, or “breathing difficulties using the stairs when having asthma”. Another description of this term was the probability of becoming ill if one did not visit a doctor regularly (ID_14).

Regarding the health-related tasks addressed in some items (e.g., items 23, 27, and 36), respondents had difficulties giving examples of their own experience. When they were asked to give an example for “*Angebote zur Gesundheitsförderung”* (efforts to promote health; item 36), they mostly thought of hygienic preventive measures at school/in the workplace, such as “washing one’s hands”, “brushing teeth,” or an ergonomically equipped workplace/school. The statements of two respondents expressed noted a lack of personal experience: they were not yet working, and a school, in their opinion, was “a healthy place”, which is why they saw no need for health promotion. Other incorrect examples given were “First Aid Course”, “drug prevention classes”.

The specific probes showed that the terms “*Wohnumgebung* (neighborhood; item 34)”, *“Wohnverhältnisse”* (housing conditions; item 42), and “*Lebensverhältnisse”* (living conditions; item 46) were fairly broad and understood in different ways by the respondents. For example, the term “housing conditions” (item 42) was defined as conditions connected to the flat/house respondents lived in (“light conditions”, “flat size”, “equipment”, “cleanliness”). Other respondents understood the term as a condition of the spatial environment, related to where one lives (“living place”, “part of the town”) or as a social dimension of living together (“neighbors”, “persons with whom one lives”).

### Challenging level of abstraction (comprehensibility)

In several items of the health promotion subscale, respondents had to establish a link between their idea of health and the following complex concepts: their living environment (item 34 and 41), political changes (item 35), housing conditions (item 42), everyday behaviors (item 43), and living conditions (item 46). In order to give a valid response, the respondent needed to know the meaning of the specific terms, to connect this meaning to their personal life and to abstract how it affects their health.

The complexity and difficulty of these tasks are well illustrated by the example of item 35. Firstly, the term “*politische Veränderungen* (political changes)” was not clear to the respondents, nor were the examples given in the German version of the item (“legislation, new preventive programs, a change of government, health care reform”). Secondly, most respondents, when asked to name “a political change” that impacted someone they had known, were not able to give an example (12 out of 20). Thirdly, several respondents (5/20) expressed difficulties understanding the connection between health and political changes:

ID_10: “To what extent does a change of government affect my well-being? (...) I mean, if now they do not change any laws (…) I don’t understand that.”

Other adolescents reported not having any interest in political issues:

ID_08: “I do not really know much about the legislation on health or any health reforms, or anything, so I never really get involved with it, but I think, it would be not so difficult to find out about it, if you just sit down and do a search somewhere”.

ID_12: “When we talk about politics at school, I really do not care about it”.

Comparable results were found concerning the connection between housing conditions and their impacts on respondents’ health (item 42). The term “*Wohnverhältnisse* (housing conditions)” was defined heterogeneously, as described above. Though some of the respondents were able to comprehend the connection between “housing conditions” and “health”, this specific topic barely seemed related to their lives:

ID_04: “I just do not think about it.”

Similarly, some of the respondents found it “difficult” for young people their age to make a statement on the matter.

### Lack of experience and knowledge (relevance)

The respondents had limited or no experience in managing some of the health-related tasks addressed by the items. For example, this was the case for items 11, 23, and 27. Though most respondents had an idea of what “*zweite Meinung von einem anderen Arzt*” (a second opinion from another doctor, item 11) meant, only a few respondents had experienced such a situation themselves. Instead, the participants answered the question with respect to what they would do in such a case, or in what type of situation they would need to contact another doctor. However, twelve out of twenty respondents found it “fairly easy” or “very easy” to judge when they needed to get a second opinion from another doctor. Further, two respondents mistook the action of getting a “second opinion” for getting a referral.

ID_10: “When I go to the physician and he is telling me to go to a surgeon or to a dermatologist, or physiotherapist - I have experienced it all.”

Approximately half of the respondents (9/20) did not believe (7/9) or did not know (2/9) whether their friends of the same age had ever gotten a “second opinion from another doctor”.

Probes on items 23 and 27 revealed little experience or specific knowledge about “*Vorsorgeuntersuchungen* (health screenings)” among the respondents. Most adolescents were able to explain this term. However, four respondents did not distinguish this type of medical examination from other common examinations that are conducted because of initial symptoms of illness, because of health problems, or in order to clarify the self-diagnosis. Not all respondents had a clear idea of which disease could be detected with the health screenings given as hints (“cancer screening, blood glucose test, blood pressure”). Cancer screenings were the best-known health screenings, followed by the blood glucose test. However, the adolescents had difficulties giving an example for a disease linked to blood pressure. Only two respondents gave an example of the recommended screenings or preventative measures related to their age, e.g., “vaccination against cervical cancer”.

Another item related to experiences in health care is item 10 (… to judge the advantages and disadvantages of different treatments). Although this item was not tested using specific probes, the interviewers noticed that more time was needed to answer the item compared with other items. Two respondents made spontaneous remarks about not having the experience or appropriate skills to perform this task.

### Mapping the response options

Even though respondents admitted to knowing only little about certain health-related topics, or to having little experience related to these topics, they estimated their ability to access, understand, judge, and apply health-related information as sufficient (“fairly easy” or “very easy”) (Table 3). In particular, searching for health information (e.g., items 1, 3 and 33) was perceived by adolescents as easy because of easy access to the Internet:

**Table 3:** Case examples of mismatches between selected response and revealed abilities in the cognitive interviews conducted in Germany (12/2015–03/2016)

Ability addressed in the item		Revealed ability using specific probes	Selected response category^a^	“Problematic” issues
Access	**Item 1** …to find information about “symptoms of illnesses”	ID_14 reported not knowing the meaning of “symptoms of illnesses”.	“fairly difficult”	Respondent answered the item without knowing the meaning of a term.
		ID_17 asked what “symptoms of illnesses” means.	“fairly difficult”	
Understand	**Item 6** …to understand leaflets	ID_07: “I don’t read leaflets but if I did, I would understand them”.	“fairly easy”	Discrepancy between reported knowledge/experience and self-estimated ability
		ID_12 reported that he had never read a leaflet completely. He had rather listened to what the doctor said.	“fairly easy”	
Judge	**Item 11**…to judge when you may need to get a second opinion from another doctor	ID_09 confirmed difficulties in judging whether a doctor counseled someone wrong when a person is not familiar with the topic. He visited another doctor only once or twice.	“fairly easy”	Discrepancy between reported knowledge/experience and self-estimated ability
		ID_17 reported getting a second opinion “not often”.	“fairly easy”	
	**Item 28** …to judge if the information on health risks in the media is reliable	ID_02 doubted being able to easily judge information in the media.	“fairly easy”	
Apply	**Item 47**… to take part in activities that improve health and well-being in your community	ID_06 gave “charitable donation” as only one example of “activities improving health”. His unique experience referred to a “charity run” that he took part in.	“very easy”	Term/item misunderstood, discrepancy between reported knowledge/experience and self-estimated ability
		ID_16 did not understand the item, described the “activities improving health” as a type of experience. The respondent was not able to understand how this type of experience is connected to health.	“fairly easy”	
		ID_17 interpreted the term “activities” as an action relating to a person, for example giving advice to a friend about exercising if he were “too fat”.	“very easy”	

^a^self-estimated ability

Results: cognitive interviews with adolescents living in Berlin (Germany) conducted in the MOHLAA study

ID_07: “I was thinking about Google, you can just google many things”.

ID_08: “Before I went to the doctor, I looked on the Internet first; because (…) I think it will be the easiest way. There (…) you put in a symptom and then it spits something out to you”.

When respondents reported not knowing a term, or when they misunderstood a question, they usually gave a response instead of skipping the question. In such cases, other respondents chose the response option “fairly difficult,” as shown by the first two examples in Table 3.

A further issue discovered through the cognitive interviews was the poor ability of participants to evaluate the “trustworthiness” of health information in the media. Our respondents almost exclusively provided proxy, secondary indicators, such as “reliability of the information source” or “reputation of the media source” based on preconceived opinions or generalizations. They also mentioned particular information sources such as “television”, “websites without advertisement” or “science press” as sources they usually trusted, or as information sources they would not trust.

ID_01: “I would not at all look for this on the internet, maybe in case of something harmless…”.

ID_08: “…also something like the yellow press, I would not trust (…), but I trust just the public news service or something more serious, or any medical or scientific websites, or magazines… I would trust it rather than any gossip-press.”

However, it was not possible to verify to what extent they were really capable of appraising sources of health information in terms of their cognitive age-related developmental stage. These demanding competencies were even questioned by the adolescents themselves: “Not at all, it is too difficult.”(ID_11).

The responses “very difficult” or “fairly difficult” were ticked when respondents did not know how to appraise the reliability of information (4/20), as well as in the opposite case, when they seemed to have critical thinking skills and recognized the difficulty of the task (2/20).

### Importance of parents

Moreover, adolescents often reported that their parents judged the reliability of information, explained the content of information (e.g., leaflets), and made health-related decisions about vaccinations and nutrition for them. They also reported turning to their parents when they felt sick or stressed.

Interviewer: “…if something was reported in the media, for example about vaccination or something similar, would you do it?”

ID_06: “Yes, if my mother would say that it is okay, I would do that”.

Interviewer: “But, I mean…you don’t look for any information on the Internet then, do you? …if you’re not well…?”

ID_06: “No, I trust my Mum”.

The adolescents relied on their parents’ knowledge and abilities and did not question their competencies.

## Discussion

The study showed that adolescent respondents had difficulties in completing the HLS-EU-Q47-GER when they were unfamiliar or inexperienced with the topics addressed. We identified unknown terms and heterogeneous interpretations of the same terms. The HLS-EU-Q47 was originally designed based on a relational concept of health literacy and focuses highly on interaction and application; however this may be problematic due to lack of experience within this particular age group. As adolescents have limited or no experience related to items on the health care and disease prevention, it is very difficult for them to realistically evaluate the required health literacy skills. The items assessing participants’ own abilities to judge and apply information about the effects of different settings on health proved to be too demanding for adolescents, or they were perceived as not interesting or as irrelevant for their daily lives.

Despite not knowing a specific term or misunderstanding the content of an item, adolescents still ticked a response rather than not responding, or they ticked the response “very/fairly difficult”. As a result, ticking such response options may reflect difficulties in understanding the item wording in some cases, rather than just respondents’ perceived difficulty in managing a health-related task. Therefore, the extent to which the self-estimated difficulty of the addressed health-related tasks reflects the knowledge of the terms used in the questionnaire remains unclear. However, the critical issue we observed was that these responses did not clearly indicate lower health skills. Even when respondents ticked the option “difficult”, further clarification would be required to determine whether the respondents would give a different answer if the items’ wordings were replaced with familiar and simpler terms.

All mentioned issues may lead to biased responses and jeopardize the validity and reliability of the questionnaire when applied among adolescents. Hence, we conclude that the questionnaire in its current form is only partly appropriate for adolescents aged 14 to 17. Several terms are subject to misinterpretation or problems of comprehension and thus fail to provide valid data. Lastly, our interviews verified the importance of interpersonal agents, especially parents, for making health-related decisions and interpreting health information. Parents were also mentioned to be the most reliable source of information.

### Impact of respondents’ experiences on the reliability and validity of responses

Our findings regarding adolescents’ prior experience and their assessments of the difficulty of certain tasks are consistent with the findings of a study by Röthlin et al. [40]. In that study, conducted with 15 year-old adolescents, a weaker correlation was found between functional health literacy, as assessed by the Newest Vital Sign [2, 57], and their subjective health literacy score measured by the HLS-EU-Q47, as compared to a similar study in adults. This finding was explained by adolescents’ limited experience with decision-making: On the one hand, adolescents are less frequently exposed to health decision-making due to their better overall health status; on the other hand, most of the important decisions at this age are made by parents or legal guardians. In comparison with older age groups, adolescents may judge the complexity and difficulty of such situations more based on expectations and much less on experience [40].

A methodological study (*n* = 188) with younger (aged 11–13) and older adolescents (aged 16–18) by Diersch and Walther suggested that younger respondents hardly seemed to have strong attitudes and concrete experience, especially towards some abstract topics they could not relate to, such as environmental protection. This may lead to a stronger orientation within the context of the questionnaire whilst answering [58]. The study indicated that particularly in the younger age group (11–13), the absence of strong attitudes towards a certain topic was expressed in the tendency toward socially desirable answers.

An association between lack of experience and biased responses was also observed in a study with adults (aged 18 years and older) by Gerich et al. [59]. The Austrian study aimed to investigate individual experiences and factors that are associated with high and low values of self-assessed health literacy, as measured by the short version (HLS-EU-Q16). Gerich et al. claimed that potential difficulties in performing health-related tasks are sometimes not recognized because respondents are not aware of the challenges related to a task. Thus, such hypothetical tasks tend to be judged as easy. In contrast, greater reflection upon health issues or more health-related experience may lead to lower scores on the HLS-EU-Q, much like a lack of general knowledge of how to address a certain situation can lead to low scores [59].

### Biased answers towards overestimation

We have interpreted the inconsistencies between difficulties expressed during probes and responses given in the questionnaire (“fairly easy” and “very easy”) as instances of overestimation. The “positive” self-estimation of health literacy by our respondents can be explained by the nature of self-report instruments. The problem of discrepancy between self-reported perceived health literacy and the objective estimation of health literacy with performance-based instruments was already discussed by Frisch et al. [60]. Screening questions, such as those of the HLS-EU-Q47, are based on self-reports, reducing the likelihood of people feeling ashamed and embarrassed when their health literacy abilities are tested directly by a performance test. The major weakness of self-estimation questions is that they are likely to assess self-efficacy or behavior instead of health literacy [60]. The question that arises from our study–what assessment approach (self-estimation versus performance-based instruments) is more appropriate for our target group?–requires further methodological studies. The current state of research on health literacy in childhood and adolescent indicates that both methods are useful and should be used complementarily in a mixed-mode approach [22].

Biased answers could also be caused by the design of the current study, particularly the interview situation [58] or the social context of the interview [61]. Those factors could explain the finding that the majority of the respondents reported not having experience with mental problems, which is a rather socially desirable answer to this sensitive topic. It is possible that participants would give more “honest” answers if they answered the questionnaire anonymously and did not need to discuss the topics with the interviewers. However, we instructed our respondents in the introduction of each interview that the interview was not concerned with the answers to the questions on the tested questionnaire but rather about how they understand these questions and how they arrived at their answers. We explained that if they did not want to answer, they did not have to do so and that if they had difficulties in answering a few questions, then we need to improve the questionnaire. Eventually, it is also probable that they gave biased answers to avoid the negative feeling caused by conceding their “incompetence” in understanding or in judging health-related information. Similar observations were made by Joffer et al. in terms of answering a question about self-reported health [62].

### Key role of interpersonal agents

Another important issue emerging from our cognitive interviews is the role of interpersonal agents. Parents were often named as the main source of health information or as those who verify the reliability of such information. Parents strongly support or make important health decisions; for example, they decide for their children what vaccinations or medical examinations will be necessary. This finding is in line with the results of recent studies indicating that parents remain the most frequently turned-to source of health information [19, 63, 64]. As an information source, the Internet was mentioned by adolescents in second or third place after, e.g., “doctors and nurses”. This may indicate that the Internet is not replacing interpersonal sources but rather supplementing them [19]. Consequently, a self-reported assessment of the health literacy competencies of adolescents should take into account their perception of the possibilities of their social context, depicted as “interpersonal factors” by Higgins et al. [65].

Our cognitive interviews verified the findings of previous studies [19, 66–68] that adolescents start their searches for health information with the search engine Google and often select the first search result instead of considering additional search results. These findings are consistent with a qualitative study by Cusack et al. [64], which showed that adolescents (aged 12–15) tended to rely only on substitute indicators, such as endorsements, when evaluating the credibility of claims. This could be explained by the finding that cognitive abilities such as information processing, logical thinking, judgment, and decision-making processes are still developing in adolescence [11]. We also supposed that adolescents are not sufficiently informed about the importance of evaluating the credibility of information and how they should do so.

These findings indicate, on the one hand, a particular need for the accurate evaluation of critical health literacy skills among adolescents, and, on the other hand, the responsibility of all health information providers and the key role of parents in providing reliable and comprehensible health information or making sound decisions for their children. Therefore, health literacy interventions should also target parents, and other significant adults in adolescents’ lives to support their critical health literacy skills.

### Methodological considerations

Referring to the method of CI by applying, e.g., the think-aloud technique, “reactive thinking” may affect the cognitive process that determines what people report on [69]. Cognitive interviews may flag “problems” that would not turn out to be “real” in surveys (errors of commission). Furthermore, cognitive interviews can fail to identify problems that exist in an actual survey (errors of omission), and findings may be inconsistent when conducted by independent groups of researchers [50]. In our cognitive interviews, we focused on the items that, based to our inclusion criteria, were identified as difficult to understand. We mostly used retrospective verbal probing instead of a think-aloud technique, which may be a more suggestive approach by flagging problems that individuals have with a questionnaire. However, this approach is recommended in the literature for our target group [61], and it ensures greater objectivity in conducting cognitive interviews through standardized probes. To minimize interpretation bias in the analyzed data, we used double coding of randomly selected parts of the data. However, because this procedure is time- and labor-intensive, we double coded approximately 20% of the transcripts (3 of 15 extensively tested items).

Another limitation relates to the characteristics of our sample. We considered only age, gender and educational level when selecting participants. No data were collected regarding the proficiency level of German language skills or on participants’ migrant backgrounds, which may be additional important factors influencing the comprehension of questionnaires measuring health literacy [29, 70, 71].

Due to the limited sample size, we could not examine whether there were any different response patterns within participating age groups and educational levels. Further, not all items could be tested with specific probes because of the time constraints generally suggested for cognitive interviews [46, 47] and for qualitative interviews with children and adolescents [72, 73]. For the same reason, it was not possible to examine how respondents justified their decisions for a certain response category for each item. Such probes could provide deeper insights into the reliability of answers and could reveal possible reasons for overestimation of health literacy skills in adolescents. Nevertheless, we are confident that we have identified major challenges that adolescents face when answering the HLS-EU-Q47-GER. In line with other studies among young respondents [51, 52], our study confirmed that adolescents are able to handle the demands of the cognitive interview and do provide valuable information that is necessary for improving a questionnaire.

Lastly, when considering change of the questionnaire scales towards more easy application among youth, it should still considered how to secure the scales’ ability to discriminate the levels of health literacy with respect to the primary aim of the questionnaire.

## Conclusions

The results of our research indicate that the applicability of the HLS-EU-Q47-GER as measurement of general health literacy among adolescents aged 14–17 is limited. Based on our findings, we assume that the data on general health literacy collected with this questionnaire are partly biased in this age group, with a tendency toward overestimation. Therefore, when using this questionnaire across all age groups, the data gathered from adolescents should be interpreted with caution.

Some of these items could be altered by adding examples and situations to which youth can easily relate or by simplifying the wording. We suggest either concretizing items with a high level of abstractions using age-related examples or omitting these items. Some of the HLS-EU-Q47 items worked as intended and can be applied among adolescents.

Our study highlights the need for age-adjusted assessment tools for health literacy that are better tailored to adolescents’ developmental stage, interests, experience, rights and responsibilities in dealing with health-related information. The required health literacy skills change depending on the phase of life, context, or individual needs; some of these skills only become relevant in adult life.

## Additional files

Additional file 1: **Table S1.** German version of the European Health Literacy Survey Questionnaire tested in the MOHLAA study in Germany (12/2015–03/2016). (DOCX 54 kb)

Additional file 2: **Table S2**. Characteristics of the participants of cognitive interviews in the MOHLAA study in Germany (12/2015–03/2016). (DOCX 45 kb)

Additional file 3: **Table S3.** Results of cognitive interviews in the MOHLAA study in Germany (12/2015–03/2016) and derived implications per individual item. (DOCX 29 kb)

## Abbreviations

CI: Cognitive interviewing
HLS-EU-Q47: The European Health Literacy Survey Questionnaire
HLS-EU-Q47-GER: The European Health Literacy Survey Questionnaire – German Version
MOHLAA: Measurement of Health Literacy Among Adolescents

## Footnotes

1 IDs refer to individual participants
