# Supplementary material for: Do adolescents understand the items of the European Health Literacy Survey Questionnaire (HLS-EU-Q47) – German version? Findings from cognitive interviews of the project “Measurement of Health Literacy Among Adolescents” (MOHLAA) in Germany
Source: Arch Public Health. 2018 Jul 10;76:46. doi: 10.1186/s13690-018-0276-2 (PMC6040081; doi:10.1186/s13690-018-0276-2)
Supplement: Supplementary file 1 — Table S1. German version of the European Health Literacy Survey Questionnaire tested in the MOHLAA study in Germany (12/2015–03/2016). (DOCX 54 kb) [file 13690_2018_276_MOESM1_ESM.docx]

**Additional file 1**

**Table S1**

**German version of the European Health Literacy Survey Questionnaire tested in the MOHLAA study in Germany (12/2015-03/2016)**

| Eingangstext | | Auf einer Skala von sehr einfach bis sehr schwierig: Wie einfach ist es Ihrer Meinung nach: |
| --- | --- | --- |
| Antwortkategorien | | 1 Sehr einfach; 2 Ziemlich einfach; 3 Ziemlich schwierig; 4 Sehr schwierig |
| **Nr.** | **HLS-EU-Q Dimension** | **Subskala: GESUNDHEITSVERSORGUNG** |
| **1*** | Finden | **…Informationen über Krankheitssymptome, die Sie betreffen, zu finden?** |
| 2 |  | …Informationen über Therapien für Krankheiten, die Sie betreffen, zu finden? |
| **3*** |  | **…herauszufinden, was im Fall eines medizinischen Notfalls zu tun ist?** |
| 4 |  | …herauszufinden, wo Sie professionelle Hilfe erhalten, wenn Sie krank sind?  (Hinweis: z.B. Arzt, Apotheker, Psychologe) |
| 5 | Verstehen | …zu verstehen, was Ihr Arzt Ihnen sagt? |
| **6*** |  | **…die Packungsbeilagen/Beipackzettel Ihrer Medikamente zu verstehen?** |
| **7*** |  | **…zu verstehen, was in einem medizinischen Notfall zu tun ist?** |
| 8 |  | …die Anweisungen Ihres Arztes oder Apothekers zur Einnahme der verschriebenen Medikamente zu verstehen? |
| 9 | Bewerten | …zu beurteilen, inwieweit Informationen Ihres Arztes auf Sie zutreffen? |
| 10 |  | …Vor- und Nachteile von verschiedenen Behandlungsmöglichkeiten zu beurteilen? |
| **11*** |  | **…zu beurteilen, wann Sie eine zweite Meinung von einem anderen Arzt einholen sollten?** |
| **12*** |  | **…zu beurteilen, ob Informationen über eine Krankheit in den Medien vertrauenswürdig sind? (Hinweis: Fernsehen, Internet oder andere Medien)** |
| 13 | Anwenden | …mit Hilfe der Informationen, die Ihnen der Arzt gibt, Entscheidungen bezüglich Ihrer Krankheit zu treffen? |
| 14 |  | …den Anweisungen für die Einnahme von Medikamenten zu folgen? |
| 15 |  | …im Notfall einen Krankenwagen zu rufen? |
| 16 |  | …den Anweisungen Ihres Arztes oder Apothekers zu folgen? |
|  |  | **Subskala: KRANKHEITSPRÄVENTION** |
| 17 | Finden | …Informationen über Unterstützungsmöglichkeiten bei ungesundem Verhalten, wie Rauchen, wenig Bewegung oder zu hohem Alkoholkonsum, zu finden? |
| **18*** |  | **…Informationen über Unterstützungsmöglichkeiten bei psychischen Problemen, wie Stress oder Depression, zu finden?** |
| 19 |  | …Informationen über empfohlene Impfungen und Vorsorgeuntersuchungen zu finden?  (Hinweis: Krebsfrüherkennung, Blutzuckertest, Blutdruck) |
| 20 |  | …Informationen darüber zu finden, wie man bestimmte Gesundheitsrisiken vermeiden oder damit umgehen kann, wie Übergewicht, hoher Blutdruck oder hoher Cholesterinspiegel? |
| 21 | Verstehen | …Gesundheitswarnungen vor Verhaltensweisen wie Rauchen, wenig Bewegung oder übermäßiges Trinken zu verstehen? |
| 22 |  | …zu verstehen, warum Sie Impfungen brauchen? |
| **23*** |  | **…zu verstehen, warum Sie Vorsorgeuntersuchungen brauchen?  (Hinweis: Krebsfrüherkennung, Blutzuckertest, Blutdruck)** |
| **Nr.** | **HLS-EU Dimension** | **Subskala: KRANKHEITSPRÄVENTION** |
| 24 | Bewerten | …zu beurteilen, wie vertrauenswürdig Gesundheitswarnungen sind, z.B. Warnungen vor Rauchen, wenig Bewegung oder übermäßigem Trinken? |
| 25 |  | …zu beurteilen, wann Sie einen Arzt aufsuchen sollten, um sich untersuchen zu lassen? |
| 26 |  | …zu beurteilen, welche Impfungen Sie eventuell brauchen? |
| 27 |  | …zu beurteilen, welche Vorsorgeuntersuchungen Sie durchführen lassen sollten?  (Hinweis: Krebsfrüherkennung, Blutzuckertest, Blutdruck) |
| **28*** |  | **…zu beurteilen, ob die Informationen über Gesundheitsrisiken in den Medien vertrauenswürdig sind? (Hinweis: Fernsehen, Internet oder andere Medien)** |
| 29 | Anwenden | …zu entscheiden, ob Sie sich gegen Grippe impfen lassen sollten? |
| 30 |  | …aufgrund von Ratschlägen von Familie und Freunden zu entscheiden, wie Sie sich vor Krankheiten schützen können? |
| 31 |  | …aufgrund von Informationen aus den Medien zu entscheiden, wie Sie sich vor Krankheiten schützen können?  (Hinweis: Zeitungen, Broschüren, Internet oder andere Medien) |
|  |  | **Subskala: GESUNDHEITSFÖRDERUNG** |
| 32 | Finden | …Informationen über gesundheitsfördernde Verhaltensweisen, wie Bewegung und gesunde Ernährung, zu finden? |
| **33*** |  | **…Informationen über Verhaltensweisen zu finden, die gut für Ihr psychisches Wohlbefinden sind?**  **(Hinweis: Meditation, körperliche Bewegung, Spazierengehen, Pilates etc.)** |
| **34*** |  | **…Informationen zu finden, wie Ihre Wohnumgebung gesundheitsförderlicher werden könnte?  (Hinweis: Minderung der Lärm- und Schadstoffbelastung; Schaffung von Grünflächen, Freizeiteinrichtungen)** |
| **35*** |  | **…etwas über politische Veränderungen herauszufinden, die Auswirkungen auf die Gesundheit haben könnten?  (Hinweis: Gesetzgebung, neue Vorsorgeprogramme, Regierungswechsel, Gesundheitsreformen etc.)** |
| **36*** |  | **…sich über Angebote zur Gesundheitsförderung am Arbeitsplatz zu informieren?** |
| 37 | Verstehen | …Gesundheitsratschläge von Familienmitgliedern oder Freunden zu verstehen? |
| 38 |  | …Angaben auf Lebensmittelverpackungen zu verstehen? |
| 39 |  | …Informationen in den Medien darüber, wie Sie Ihren Gesundheitszustand verbessern können, zu verstehen? (Hinweis: Internet, Zeitungen, Zeitschriften) |
| 40 |  | …Informationen darüber, wie Sie psychisch gesund bleiben können, zu verstehen? |
| 41 | Bewerten | …zu beurteilen, wie sich Ihre Wohnumgebung auf Ihre Gesundheit und Ihr Wohlbefinden auswirkt?  (Hinweis: Ihre Gemeinde, Ihre Nachbarschaft) |
| **42*** |  | **…zu beurteilen, wie Ihre Wohnverhältnisse dazu beitragen, dass Sie gesund bleiben?** |
| 43 |  | …zu beurteilen, welche Alltagsgewohnheiten mit Ihrer Gesundheit zusammenhängen? (Hinweis: Trink- und Essgewohnheiten, Bewegung etc.) |
| 44 | Anwenden | …Entscheidungen zu treffen, die Ihre Gesundheit verbessern? |
| 45 |  | … einem Sportverein beizutreten oder einen Sportkurs zu belegen, wenn Sie das wollen? |
| **46*** |  | **…Ihre Lebensverhältnisse, die Auswirkungen auf Ihre Gesundheit und Ihr Wohlbefinden haben, zu beeinflussen? (Hinweis: Trink- und Essgewohnheiten, Bewegung etc.)** |
| **47*** |  | **…sich für Aktivitäten einzusetzen, die Gesundheit und Wohlbefinden in Ihrer Umgebung verbessern?** |

*Items tested with specific probes are shown in bold.
The H LS-EU-Q47-GER is a translation into German of the English version of the HLS-EU-Q47 that has been verified within the scope of the European Health Literacy Survey [35].
