# Supplementary material for: Do adolescents understand the items of the European Health Literacy Survey Questionnaire (HLS-EU-Q47) – German version? Findings from cognitive interviews of the project “Measurement of Health Literacy Among Adolescents” (MOHLAA) in Germany
Source: Arch Public Health. 2018 Jul 10;76:46. doi: 10.1186/s13690-018-0276-2 (PMC6040081; doi:10.1186/s13690-018-0276-2)
Supplement: Supplementary file 2 — Table S2. Characteristics of the participants of cognitive interviews in the MOHLAA study in Germany (12/2015–03/2016). (DOCX 45 kb) [file 13690_2018_276_MOESM2_ESM.docx]

**Additional file 2**

**Table S2
Characteristics of the participants of cognitive interviews in the MOHLAA study in Germany (12/2015-03/2016)**

|  |  | **Education level** | | | |  |
| --- | --- | --- | --- | --- | --- | --- |
|  |  | **Not in high school** | | **High school** | |  |
|  | **Sex** | Girls | Boys | Girls | Boys |  |
| **Age** | 14 y. | 1 | 3 | 1 | 1 | **6** |
|  | 15 y. | 1 | 3 | 1 | 0 | **5** |
|  | 16 y. | 2 | 2 | 1 | 0 | **5** |
|  | 17 y. | 1 | 1 | 1 | 1 | **4** |
|  |  | 5 | 9 | 4 | 2 |  |
| Total | | **14** | | **6** | | **20** |

The category “not in high school” included all types of lower- and middle-secondary
level schools existing in Berlin’s education system (Germany) at the time.
