# Supplementary material for: Do adolescents understand the items of the European Health Literacy Survey Questionnaire (HLS-EU-Q47) – German version? Findings from cognitive interviews of the project “Measurement of Health Literacy Among Adolescents” (MOHLAA) in Germany
Source: Arch Public Health. 2018 Jul 10;76:46. doi: 10.1186/s13690-018-0276-2 (PMC6040081; doi:10.1186/s13690-018-0276-2)
Supplement: Supplementary file 3 — Table S3. Results of cognitive interviews in the MOHLAA study in Germany (12/2015–03/2016) and derived implications per individual item. (DOCX 29 kb) [file 13690_2018_276_MOESM3_ESM.docx]

**Additional file 3**

**Table S3
Results of cognitive interviews in the MOHLAA study in Germany (12/2015-03/2016) and derived implications per individual item**

| **No.** | **Results relating to the single item** | **n** | **miss.** | **Implications for the MOHLAA questionnaire** |
| --- | --- | --- | --- | --- |
| **1*** | The term “*Krankheitsymptome* (symptoms of illnesses)” is unknown to some adolescents (5/20) (C3). | 18 | 2 | Explain the term “symptoms of illnesses” or replace it with another, simpler word |
| 2 | No relevant remarks | 19 | 1 | Adaptation not required |
| **3*** | The item was understood well. Respondents were able to recall either experience or knowledge about a medical emergency, on which they could elaborate. Three respondents were unsure to whom the case of medical emergency relates: themselves or another person (ID_12, ID_14 and ID_18). ID_10 remarked that “an emergency case” can be broadly defined. | 20 | 0 | Give examples of emergency situations, e.g., accident, severe burns, alcohol poisoning that are relevant for this age group. |
| 4 | No relevant remarks | 20 | 0 | Adaptation not required |
| 5 | The item seems to be well understood. Two respondents noted that their answers would be dependent on whether a doctor says something clear or whether an illness is familiar to him/her. | 20 | 0 | Adaptation not required |
| **6*** | The item seems to be well understood. There were some hints that leaflets are not read completely or not read at all and that they are not well understood by adolescents (C5). The task of “understanding everything" from a leaflet was assessed as “difficult”. The majority of the respondents did not believe that friends of their age had ever read a complete leaflet. Two respondents reported that their parents usually did it for them (ID_08, ID_12). | 20 | 0 | A filter question is recommended: Have you ever tried to read a leaflet? |
| **7*** | The item was tested together with item 3. The difference between the meaning of the sentence “find out (*herausfinden*)” (Item 3) and “understand (*verstehen*) what to do in a case of medical emergency” (item 7) was not clear to respondents. Item 7 was not understood more often (C2). Three respondents claimed that both items mean the same (ID_06, ID_16, ID_17). Another three could not explain the difference between the items (ID_09, ID_14 and ID_21). “To understand” was associated with applying (ID_05, ID_01, ID_04) emergency instructions (C3). | 20 | 0 | Further clarification is necessary to what extend the item 3 and 7 differentiate from each other. |
| 8 | No relevant remarks | 20 | 0 | Adaptation not required |
| 9 | ID_02 reported that the item is worded imprecisely (C1). ID_07, ID_14 had difficulties understanding the item and were confused about which type of information from a doctor was meant (C2). ID_17 did not understand the item (C2.) The respondents assumed that doctors always provide information that applied to them as patients. | 19 | 1 | The question must be phrased more precisely. |
| 10 | Some respondents (ID_12, ID_14 and ID_17) did not understand or had difficulties understanding the meaning of the item (C2). ID_04, ID_05 reported that they have had no experience relating to the topic. ID_04 doubted that young people are capable of “judging the advantages and disadvantages of different treatment options” (C5). | 19 | 1 | A filter question is recommended, e.g., Have you ever had to choose between different treatment options?  No significant relevance for the age-group. |
| **11*** | “*Zweite Meinung* (to get a second opinion)” was described correctly by the majority of the respondents. However, some participants had difficulties explaining the term or did it incorrectly (ID_07, ID_11, ID_06, ID_10 and ID_12), and two participants interpreted the term as a referral (C2, C3). There were a few hints that adolescents have had no or little experience with performing this health care task (C5). | 20 | 0 | No significant relevance for the age-group. |
| **12*** | The item was perceived as “too long”, apparently due to the additional hint (C1). The German term “*vertrauenswürdig* (reliable, trustworthy)” was well understood by almost all of the respondents. There were some comments that parents usually verify the reliability of information and that adolescents are not capable of verifying the reliability of information by themselves (C5); nevertheless, they ticked the responses: “very easy” or “easy” (C6). The reliability of their answers was assessed using the probe: “How can you recognize whether [health] information in the media is reliable?” Their responses indicated that adolescents used only some kind of proxy, secondary criterion” e.g., type of media. | 19 | 1 | Simplify wording of the item. |
| 13 | One respondent was unsure of the intention of the question. The interviewers observed uncertainty (hesitating, long thinking) when two respondents answered the question (C2). | 20 | 0 | No significant relevance for the age-group. Further clarification will be necessary to determine the extent to which adolescents make decisions about treatments by themselves and whether they have ever experienced such situations. |
| 14 | No relevant remarks. The distribution of responses indicates that the item may not differentiate among respondents (C4). Three respondents evaluated the item as an “easy” item. | 19 | 1 | Adaptation not required |
| 15 | ID_15 and ID_17 have never called an ambulance. ID_15 asked how to answer this question without having had this experience. Six respondents evaluated the item as an “easy” item (C4). | 20 | 0 | The item seems to be “too easy.” The item should be made more difficult, e.g., what is the emergency telephone number? |
| 16 | No relevant remarks | 20 | 0 | Adaptation not required |
| 17 | ID_15 was confused by the German term “*Unterstützungsmöglichkeiten* (possibilities of support)”. The term was not always well understood (C3). See the findings relating to item 18. | 20 | 0 | Revise the wording of the item or explain the term “possibilities of support.” |
| **18*** | For two respondents, the term “*Unterstützungsmöglichkeiten* (possibilities of support)” was unfamiliar (C3). The majority of respondents claimed to have no or only a few experiences related to mental health problems (C5). Only two respondents reported having their own experience with the topic. Others answered hypothetically. Family and friends were usually identified as a type of “supporter” or those who assist when seeking help. | 20 | 0 | See below. Provide examples of “mental health problems” common in the age group, e.g., chronic stress, depression, bullying, eating disorders. |
| 19 | Four respondents had difficulties understanding the question; one of the respondents did not answer the item (C2, C3). The term “*Vorsorgeuntersuchungen* (health screenings)” was often misunderstood. See the findings relating to item 23. | 19 | 1 | No significant relevance for the age-group. |
| 20 | Respondents ID_16 and ID_17 didn’t understand the item (C2). The term “*Gesundheitsrisken* (health risks)” was often misunderstood (C3). See the findings relating to item 28. | 20 | 0 | Replace or explain the term “health risks.” |
| 21 | No relevant remarks | 20 | 0 | Adaptation not required |
| 22 | No relevant remarks. See the findings relating to item 26. | 20 | 0 | No significant relevance for the age-group; decisions about vaccinations are usually made by their parents. |
| **23*** | The term “*Vorsorgeuntersuchungen* (health screenings)” was misunderstood (C3) by three respondents (ID_10, ID_14, ID_20). It was mistaken for an additional medical examination in order to clarify or verify a diagnosis. The respondents answered the item abstractly because they had no or little experience or specific knowledge about this type of medical examination (C5). They had difficulties giving an example of a health screening related to their age apart from cancer screening (C5). Health screenings were generally considered as something positive and necessary by adolescents (“it can be always helpful”). This attitude may indicate socially desirable answers to the item (C6). | 19 | 1 | No significant relevance for the age-group; decisions about health screenings are usually made by their parents or other adults. |
| 24 | No relevant remarks. The question was assessed by two respondents as “difficult”. | 20 | 0 | Adaptation not required |
| 25 | No relevant remarks | 20 | 0 | Adaptation not required |
| 26 | Two respondents (ID_04, ID_05) reported that they had never dealt with any issues of vaccinations because their parents decided about that (C5). This may indicate that this task [vaccination] does not belong to the competencies of adolescents. | 20 | 0 | No significant relevance for the age-group; decisions about vaccinations are usually made by their parents. |
| 27 | One respondent was confused because of the wording being similar to item 23 (C1). ID_04 and ID_12 suggested that the task seems to be too “difficult” or, due to the age of the respondents, not relevant to them. They lacked experience on judging which screenings they should have (C5). See the findings relating to item 23. | 19 | 1 | No significant relevance for the age-group; decisions about health screenings are usually made by their parents or other adults. |
| **28*** | The term “*Gesundheitsriken* (health risks)” was misunderstood or unfamiliar (6/20) (C3). Almost half of the respondents were not able to give an example of health risks among their friends or peers. The respondents seemed to be very unsure about how to judge the reliability of information in the media. | 19 | 1 | Replace the term “health risks” with a simpler word. |
| 29 | Two respondents (ID_02, ID_10) reported that had not previously known that it is possible to get a flu vaccination (C5). See the findings relating to items 22 and 26. | 20 | 0 | No significant relevance for the age-group; flu vaccination is not recommended for this age group. |
| 30 | No relevant remarks. ID_2 reported that she did not understand the intention of the question. | 19 | 1 | Adaptation not required |
| 31 | No relevant remarks | 20 | 0 | Adaptation not required |
| 32 | No relevant remarks. ID_06 did not understand the item. | 20 | 0 | Adaptation not required |
| **33*** | The term “*psychisches Wohlbefinden* (mental well-being)” was unknown or misunderstood by some respondents (6/20) (C3). Three respondents suggested that the term should be described or replaced with a simpler word. Another three respondents did not understand the item, but only two of them skipped the question (C2). The relating hints (“mediation, exercise, walking, pilates”) were hardly associated with “mental well-being”. Nevertheless, the respondents were able to give some examples of activities that they usually engage in for their mental well-being without needing to search for any related information about this topic. | 18 | 2 | Revise the term “mental well-being.” Provide age-specific examples of activities that are good for mental health and well-being. |
| **34*** | ID_01 made a remark about the length of the sentence (“too complex”) (C1). Four respondents had difficulty understanding the item (C2). The English term “neighborhood” was roughly translated as “*Wohnumgebung*”, i.e., “an area where one lives”. “*Wohnumgebung*” was variously interpreted and often misunderstood (C3). Some respondents found it “difficult” to answer the item because they had never thought about such topics (C5); other respondents found it “easy” because finding any information is generally easy for them (C6). The link between “*Wohnumgebung*” and “health” was completely unclear to one respondent. | 17 | 3 | The item does not seem to be appropriate for this age group. Reduce the level of abstraction of the item. |
| **35*** | The term “*politische Veränderungen* (political changes)” and the related hints were unfamiliar or unknown to the majority of the respondents (C3). The link to “health” was not clear to five respondents (C2). Two respondents did not understand the item correctly (C2). There were some hints that adolescents did not have any interest in political topics (C5), however, seeking information seemed to be easy to them. Interestingly, half of the respondents evaluated the item as “easy” to answer. | 19 | 1 | The item does not seem to be appropriate or relevant for this age group. |
| **36*** | The term “*Angebote zur Gesundheitsförderung* (efforts to promote your health)” was often misunderstood (C3). Respondents associated the term with – or mistook it for – means of disease prevention. Five respondents could not give any example or any correct example of the term. Respondents mentioned that the term lacked relevance to adolescents’ lives because most of them are still at schools that they regarded as ”a healthy place” (C5). | 18 | 2 | Simplify the term “Angebote zur Gesundheitsförderung (efforts to promote your health)” by giving examples relating to the adolescents’ lives. |
| 37 | No relevant remarks | 20 | 0 | Adaptation not required |
| 38 | No relevant remarks | 20 | 0 | Adaptation not required |
| 39 | No relevant remarks | 20 | 0 | Adaptation not required |
| 40 | One respondent noted that he could not answer the question due to lacking experience regarding mental health (C5). Another skipped the question without any explanation. | 18 | 2 | Adaptation not required |
| 41 | One respondent reported that he didn’t understand how “*Wohnumgebung*” affects health and well-being (C2). Two respondents had difficulties understanding the terms “*Wohnumgebung*” und “*Wohlbefinden* (well-being)” (C3). See the findings related to item 34.  The translation of the item into German might slightly change the meaning of the original HLS-EU-Item. | 17 | 3 | The item is not appropriate for this age group. Reduce the level of abstraction of the item. |
| **42*** | The term “*Wohnverhältnisse* (housing conditions)” was defined heterogeneously (C3). The term was referred to as a flat, house, room or living environment, place of residence, or with whom one lives. The link between “housing conditions” and health was hard to understand for some respondents (C2). Lack of relevance to the adolescent life-world was mentioned (C5). | 20 | 0 | The item is not appropriate for this age group. Reduce the level of abstraction of the item. |
| 43 | The term “*Alltagsgewohnheiten* (everyday behavior)” was not clear for three respondents (C3). Some respondents had difficulties understanding the item, in particular the link between “health” and “every day behavior” (C2). Two respondents assessed the item as “difficult” to answer (C4). | 17 | 3 | Simplify the wording of the item.  Give examples of everyday behavior, e.g., eating, drinking,  exercises, relaxation, body care. |
| 44 | No relevant remarks. One respondent mentioned that the power to make such decisions belongs to a doctor and not to him. | 20 | 0 | Adaptation not required |
| 45 | No relevant remarks. | 20 | 0 | Adaptation not required |
| **46*** | The term “*Lebensverhältnisse* (living conditions)” was not always well understood (C3). In order to answer the item, the respondents used the hints (drinking and eating habits, exercise etc.). The hints were easily understood. The given examples for “living conditions” (C2) did not match with the related hints. However, the respondents reported that the hints helped them to understand the link between “living conditions” and “health”. The respondents tended to assess this task as “easy” because they believed that “living conditions” are easy to change, at the same time they reported lacking the motivation to do so (C5). | 18 | 2 | The item is not appropriate for this age group. Reduce the level of abstraction of the item. |
| **47*** | A few spontaneous queries, remarks or "longer thinking" of the respondents were observed. Some respondent had difficulties understanding the question. The term "activities" was not clear to some respondents (C2, C3). One respondent advised adding an explanation for the term "activities". The term "*Umgebung* (environment)" was diversely interpreted. | 16 | 4 | Simplify the wording of the item and give examples, e.g., for more parks and playing fields, less noise and traffic, better air. |

C1 - Sentence structure/grammar, C2 - Comprehensibility of item‘s content, C3 - Understanding of terms/hints, C4 - Difficulty, C5 -Experience/knowledge, C6 - Reliability of the response, C7 - Accordance of the formal response category with an internal ascertained response category. The not-fulfilled criteria are shown in brackets. miss. – number of missing values
